# The significance of metagenomic next-generation sequencing and targeted next-generation sequencing in the diagnostic evaluation of patients with suspected pulmonary infections

**DOI:** 10.3389/fcimb.2025.1552236

**Published:** 2025-06-12

**Authors:** Yucong Tang, Shujin Tang, Yingchao Zhang, Zhentao Lin, Shuxiang Shan

**Affiliations:** Department of Pulmonary and Critical Care Medicine, Tianjin Medical University Baodi Hospital, Tianjin, China

**Keywords:** mNGS, tNGS, bronchoalveolar lavage fluid, pathogens, pulmonary infection

## Abstract

**Objective:**

To investigate the diagnostic value of metagenomic next-generation sequencing (mNGS) and targeted next-generation sequencing (tNGS) in identifying pathogens in patients with pulmonary infections.

**Methods:**

A retrospective analysis was conducted on 155 patients with suspected lung infections who underwent alveolar lavage and were admitted to the Department of Respiratory and Critical Care Medicine at Baodi Hospital, Tianjin Medical University, from July 2023 to December 2023. The bronchoalveolar lavage fluid (BALF) samples obtained were subjected to mNGS, tNGS and culture methods to compare their diagnostic efficacy in identifying lung infection pathogens.

**Results:**

The results indicated that both tNGS and mNGS methods exhibit comparable detection efficiencies in identifying pathogens in patients with pulmonary infections, significantly outperforming BALF culture approach. In terms of diagnostic accuracy, tNGS exhibited a higher sensitivity than mNGS, with rates of 96.1% and 75.7% respectively (P>0.05). However, the specificity of tNGS was slightly lower than that of mNGS, with rates of 59.1% and 68.2% respectively (P>0.05). It is noteworthy that this difference in specificity was not statistically significant.

**Conclusion:**

tNGS exhibits a diagnostic efficacy comparable to mNGS, particularly in its sensitivity for identifying lung infections, as evidenced by expert insights and clinical applications. Furthermore, tNGS offers advantages in convenience, time efficiency, and cost-effectiveness, hinting at its potential to serve as an alternative to mNGS in clinical settings.

## 1 Introduction

Lung infections constitute a prevalent and substantial global health issue, imposing a considerable socioeconomic burden on societies worldwide. Early identification of the underlying pathogen is paramount for the formulation of an appropriate and effective clinical treatment plan. Traditional methods of pathogen detection, such as pathogen culture, antigen and antibody detection, and smear microscopy, each possess their unique advantages and disadvantages. However, the overall detection rates achieved through these methods remain unsatisfactory, with approximately 50% of respiratory infections remaining unidentified (Zhu et al., 2018).

In recent years, high-throughput sequencing technologies have garnered increasing attention in clinical settings due to their enhanced sensitivity and reduced detection time. Among these, metagenomic next-generation sequencing (mNGS) and targeted next-generation sequencing (tNGS) have emerged as prominent tools. mNGS possesses the capability to detect a wide array of pathogenic microorganisms, including emerging and rare species (Hilt and Ferrieri, 2022). However, mNGS also has limitations, such as lower sensitivity compared to PCR at low viral loads, inability to distinguish infecting pathogens from colonizing bacteria, and a lack of standardized reporting and interpretation criteria (Chiu and Miller, 2019; Diao et al., 2021; Liu, 2024). Currently, mNGS is typically considered when patients remain undiagnosed by traditional microbiological methods and empirical anti-infective therapy has been ineffective. On the other hand, tNGS utilizes polymerase chain reaction (PCR) technology to specifically amplify target genes, thereby minimizing interference from host nucleic acids. This method demonstrates enhanced specificity and sensitivity in detecting pathogens (Leo et al., 2017). Despite the growing utilization of mNGS and tNGS, there is a scarcity of comprehensive studies comparing their efficacy in diagnosing pulmonary infectious diseases.

The present study compared the results of mNGS and tNGS in bronchoalveolar lavage fluid (BALF) samples obtained from 155 patients with suspected pulmonary infections, primarily to evaluate their effectiveness and differences in pathogen detection in these patients. Furthermore, this study aimed to explore the respective advantages and disadvantages in the clinical application of mNGS and tNGS, thereby contributing to the ongoing advancement in the diagnosis and management of pulmonary infectious diseases.

## 2 Methods

### 2.1 Study subjects

A retrospective analysis was conducted on the clinical data of patients admitted to the Department of Respiratory and Critical Care Medicine, Baodi Hospital of Tianjin Medical University, between July 2023 and December 2023. Patients suspected of lung infections underwent BALF mNGS and tNGS, which are advanced diagnostic techniques that enable the detection of a wide range of pathogens, including bacteria, viruses, fungi, and parasites, in respiratory samples. Inclusion criteria encompassed: 1) patients admitted to the aforementioned department with suspected lung infections during the specified timeframe; and 2) patients who consented to bronchoscopy and BALF collection for mNGS and tNGS analysis. Exclusion criteria comprised: 1) individuals who did not complete bronchoscopy or failed to successfully obtain lavage fluid samples; and 2) patients with incomplete clinical data. Clinicians determine the clinical relevance of NGS-detected microorganisms (categorized as pathogenic, opportunistic, or colonizing) by integrating findings from the patient’s clinical presentation, immune status, blood biochemical parameters, imaging features, and conventional microbiological tests. Additionally, the interpretation of negative NGS results requires correlation with clinical manifestations and supplementary diagnostic evidence. The interpretation of these results was conducted by three trained physicians. This study was approved by The Ethics Committee of Baodi Hospital affiliated to Tianjin Medical University (No. BDYYLL202506) and was conducted according to the principles of the Helsinki Declaration.

### 2.2 Bronchoscope irrigation procedure

Following the insertion of the bronchoscope’s tip into the selected bronchial branch, 20–40 mL of saline was administered. The resulting BALF was then collected and transferred into a sterilized container for subsequent culture, mNGS detection, and tNGS detection. Samples were stored on dry ice for 2 hours before being dispatched to the Tianjin Kingmed Diagnostics Laboratory Co.,Ltd. for further mNGS and tNGS analysis.

### 2.3 Microbiological culture

BALF specimens were inoculated onto blood agar plates, MacConkey agar plates, Sabouraud dextrose agar plates, and chocolate agar plates using a sterile inoculation loop. Blood agar and chocolate agar plates were incubated at 35°Cunder 5% CO2 atmosphere, while MacConkey agar and Sabouraud dextrose agar plates were cultured in a conventional incubator at 35°C. Following 18–24 hours of continuous culture, bacterial growth was examined across all plates. Preliminary identification was performed based on colonial morphology and Gram-staining characteristics. Subsequent species-level identification was conducted according to the biological characteristics of each bacterial type, accompanied by antimicrobial susceptibility testing.

### 2.4 mNGS assay and report

Upon receipt, the samples underwent centrifugation and processing. Nucleic acids were extracted using the Universal DNA/RNA Extraction Kit for Environmental Samples (TR202JY-50, Jianshi Biotechnology, Beijing, China) through centrifugal column-based protocols. After qubit quantification, the nucleic acids were subjected to standardized DNA library preparation procedures. PCR amplification and sequencing on an MGISEQ-200 sequencer were then executed, and the raw sequencing data were filtered using Fastp (v0.20.0) (Chen et al., 2018) with culling the reads containing more than 40% of low-quality bases (mass value less than 15), the reads containing more than 5 n-bases, the reads matched to the sequencing primer, the PCR repeat sequence, and the human nucleic acid sequences. The non-human high-quality sequences were then compared with the microbial genome database by BWA (v0.7.17-r1198-dirty) (Li, 2013), and the results of microbial species identification were obtained. The microbial database was collected from the NCBI RefSeq and GenBank databases.

### 2.5 tNGS assay and report

After liquefying the treated samples, nucleic acid extraction and quantification were performed. Total nucleic acid extraction was performed either manually or through automation using the Nucleic Acid Extraction/Purification Kit (KS118-BYTQ-96, Guangzhou KingCreate Biotechnology Co. Ltd., Guangzhou, China) on the KingFisher™ Flex Purification Platform (Thermo Fisher Scientific, Waltham, USA). The RP100 Respiratory Pathogen Microorganisms Multiplex Testing Kit (Guangzhou KingCreate Biotechnology Co. Ltd., Guangzhou, China) on BALF were used to conduct library preparation. PCR amplification was used to enrich the target pathogens, and steps were taken to eliminate the interference from human background nucleic acid within the sample. Qualified libraries were used as input for sequencing, which was performed on the KM MiniSeqDx-CN Platform (Guangzhou KingCreate Biotechnology Co. Ltd., Guangzhou, China). The raw sequencing data were filtered with criteria consistent with mNGS to obtain the non-human high-quality sequences. The resulting data were then aligned against a reference database using Bowtie2 v2.4.1 (Langmead and Salzberg, 2012) in “very-sensitive” mode. The reference database was compiled from multiple sources, including the GenBank database, RefSeq database, and NT database from the National Center for Biotechnology Information (NCBI)

### 2.6 Statistical analysis

All statistical analyses were conducted using R software (version 4.3.1). The Wilcoxon test was used to evaluate differences between the two groups, considering P<0.05 as statistically significant. ROC curves were generated to assess the diagnostic efficacy of the assays, providing a visual representation of their sensitivity and specificity, and enabling the comparison of their diagnostic performance through the area under the curve (AUC). Count data were presented as cases or percentages (%), and the chi-squared test was used to compare the sensitivity and specificity between mNGS and tNGS.

### 2.7 Data availability

The raw sequence data reported in this paper have been deposited in the Genome Sequence Archive (Genomics, Proteomics & Bioinformatics 2021) in National Genomics Data Center (Nucleic Acids Res 2022), China National Center for Bioinformation/Beijing Institute of Genomics, Chinese Academy of Sciences (GSA: CRA021644) that are publicly accessible at https://ngdc.cncb.ac.cn/gsa (Chen et al., 2021; CNCB-NGDC Members and Partners, 2022).

## 3 Result

### 3.1 Patient characteristics

In our study, a total of 155 patients were enrolled in this analysis. Among them, 125 patients were confirmed to have pulmonary infection, while the remaining 30 patients were diagnosed with non-pulmonary infections. The demographic and clinical information of all cases between two groups were collected, including gender, age, length of stay, white blood cell count, neutrophil ratio and primary disease, as summarized in **Table 1**. We observed significant differences in length of stay, white blood cell count, and neutrophil ratio between patients with pulmonary infections and those with non-pulmonary infections, highlighting clinical distinctions between the two groups.

**Table 1:** Baseline characteristics of the 155 patients enrolled.

Characteristics		Pulmonary infection (n=125)	Non-pulmonary infection (n=30)	P
Gender	Male	83 (66.40%)	19 (63.33%)	0.75
	Female	42 (33.60%)	11 (36.67%)	
Age		54.16 ± 20.06	61.97 ± 11.02	0.17
Length of stay (day)		9.30 ± 4.90	5.40 ± 3.81	<0.001
White blood cell (×10^9^)		10.53 ± 5.53	6.48 ± 2.02	<0.001
Neutrophil ratio (%)		72.56 ± 12.92	60.56 ± 12.13	<0.001
Primary disease	Yes	71 (56.80%)	23 (76.67%)	0.05
	No	54 (43.20%)	7 (23.33%)	

### 3.2 Pathogen detection using mNGS, tNGS and culture methods

**Figure 1:**
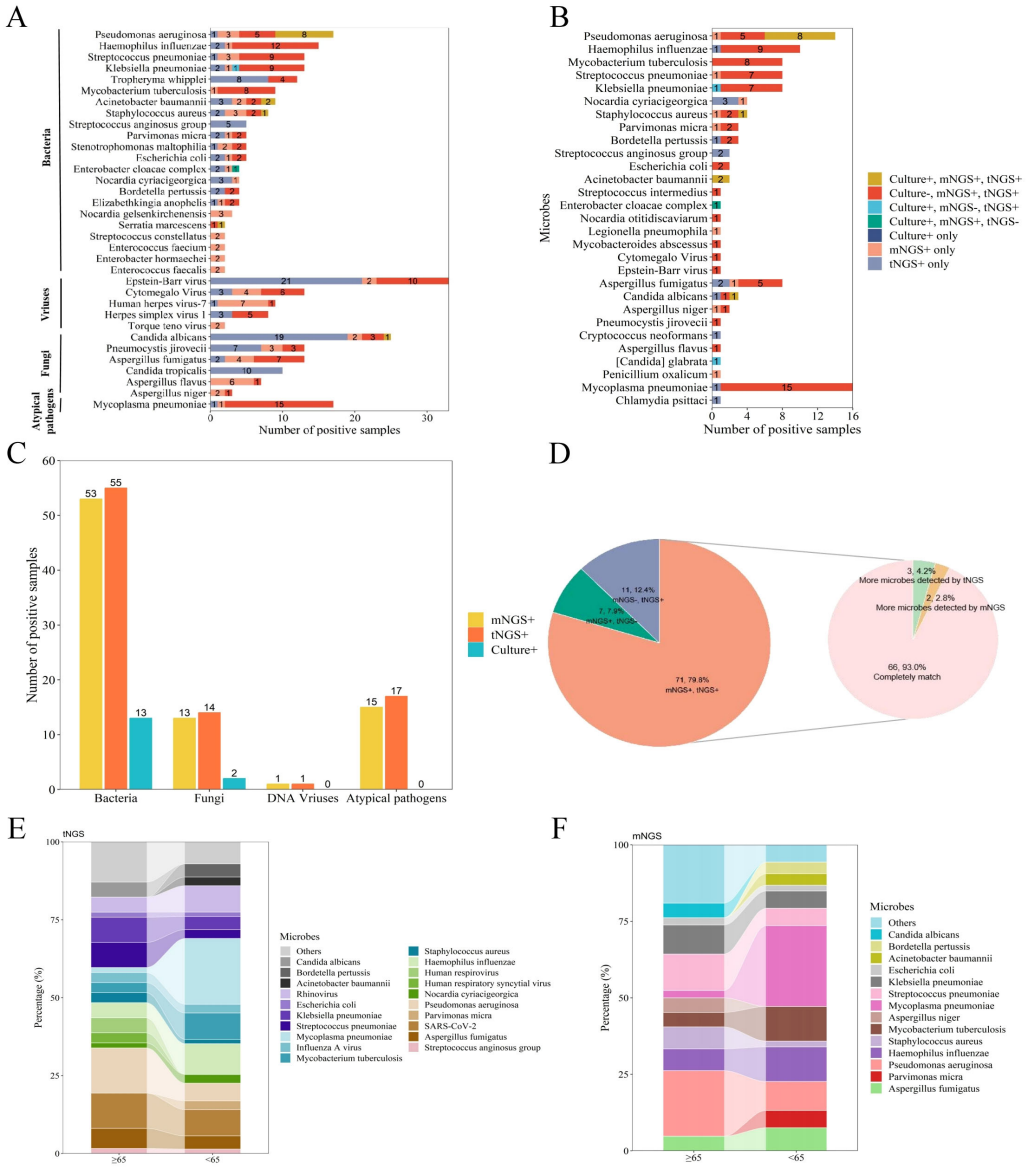
An evaluation of the efficacy in identifying pathogens utilizing mNGS, tNGS, and culture methods. **(A)** A categorization of the distribution of microorganisms detected in more than two samples through the application of mNGS, tNGS, and culture assays. **(B)** The distribution of potential pathogens and the respective contributions of tNGS, mNGS, and culture in the detection of these pathogens. **(C)** A comparison of the number of positive samples for bacteria, fungi, DNA viruses, and atypical pathogens among tNGS, mNGS, and culture methods. **(D)** A pie chart illustrating the consistency of results between mNGS and tNGS, with double positive results further categorized into completely matches, more microbes detected by mNGS, and more microbes detected by tNGS. **(E)** The variability in the distribution of pathogens across different age groups when utilizing tNGS. **(F)** The variability in the distribution of pathogens across different age groups when utilizing mNGS.

A total of 155 specimens were subjected to test through mNGS, tNGS, and BALF culture protocols. The mNGS technique identified 210 strains belonging to 49 different microorganisms, while tNGS detected 248 strains from 44 microorganisms. BALF culture, on the other hand, revealed 19 strains from 11 microorganisms. Among the bacteria, the three most frequently detected were *Pseudomonas aeruginosa* (5.2%), *Haemophilus influenzae* (4.6%), and *Streptococcus pneumoniae* (4.0%). For viral detection, the top three pathogens identified were *Epstein-Barr virus* (10.0%), *cytomegalo virus* (4.0%), and *human herpes virus 7* (2.7%). For fungal detection *Candida albicans* (7.6%), *Pneumocystis jirovecii* (4.0%), and *Aspergillus fumigatus* (4.0%) were the predominant fugal pathogens (**Figure 1A**).

Among the 125 patients diagnosed with pulmonary infections, pathogenic microorganisms were identified in 103 cases (82.4%), with 30 of these cases diagnosed as mixed infections. A total of 110 pathogens were detected among 103 patients. As demonstrated in **Figure 1B**, the majority of pathogens were successfully detected by both mNGS and tNGS, showcasing their complementary strengths in pathogen identification. In contrast, BALF culture identified fewer pathogens, indicating its limitations in sensitivity compared to the sequencing technologies. Additionally, tNGS and mNGS demonstrated similar efficacy in detecting specific microbial types and co-infection, and significantly outperformed traditional BALF culture, as shown in **Figure 1C**, **Table 2** and **Supplementary Table 1**.

**Table 2:** Positive sample detection rates for mNGS, tNGS, and culture methods across various microbial types.

Pathogen	mNGS (%)	tNGS (%)	Culture (%)
Bacteria	88.33	91.67	21.67
Fungi	81.25	87.50	12.50
Atypical pathogens	88.24	100.00	0.00
DNA virus	50.00	100.00	0.00

Furthermore, we conducted a comparative analysis to evaluate the detection rates of positive samples and the discrepancies in identifying pathogenic microorganisms using two distinct approaches. As showed in **Figure 1D**, 71 cases (79.8%) were positive with both tNGS and mNGS, while 11 cases (12.4%) were positive only with tNGS and 7 cases (7.9%) were exclusively positive with mNGS. Among patients with positive results in both tNGS and mNGS, the pathogens detected by both methods matched exactly in 66 cases (93.0%). More pathogens were detected by tNGS in three cases (4.2%), while more pathogens were detected by mNGS in two cases (2.8%). The results indicate that both tNGS and mNGS methods have comparable detection efficiencies in identifying pathogens in patients with pulmonary infections, significantly surpassing the BALF culture approach, thereby highlighting the superior performance of NGS detection methods in pathogen detection.

Concurrently, we performed a comparative analysis to examine the variations in the distribution of BALF pathogens across different age cohorts. We found that, for both mNGS and tNGS, the predominant pathogens in patients aged 65 and above were *Pseudomonas aeruginosa*, *Streptococcus pneumoniae*, *Klebsiella pneumoniae*, and *Candida albicans*. Among patients younger than 65 years, microorganisms such as *Parvimonas micra*, *Haemophilus influenzae*, *Mycoplasma pneumoniae* and *Bordetella pertussis* were more prevalent and ranked higher in the pathogen spectrum (**Figures 1E, F**). Previous studies have shown that *Klebsiella pneumoniae* and *Pseudomonas aeruginosa* in acute respiratory infections follow an age-related pattern, with the positive detection rates increasing with patient age, consistent with the findings of this study. The consistency between mNGS and tNGS in the analyzing pathogen distribution across different age groups further supports the reliability and effectiveness of both in pathogen detection.

### 3.3 Clinical evaluation of mNGS, tNGS and culture methods

To assess the clinical diagnostic performance of the three methodologies, we calculated the sensitivity, specificity, and accuracy for the different detection methods based on the clinical diagnosis of each sample, as shown in **Table 3**. Among the three detection techniques, tNGS exhibited the highest sensitivity of 96.1%, followed by mNGS with 75.7%, while culture demonstrated the lowest sensitivity at 14.6%. Conversely, the specificity trend was inverse to that of sensitivity, with culture achieving 100.0% specificity, mNGS at 68.2%, and tNGS at 59.1%, respectively. In summary, tNGS demonstrated a marginally superior clinical diagnostic performance compared to mNGS, while culture fared the worst, with F1 score of 0.830, 0.938, and 0.254, respectively. Our comparative analysis of tNGS and mNGS demonstrated a diagnostic concordance rate of 70.4% between the two methods (**Table 4**). Besides, a chi-squared test was subsequently performed to assess the sensitivity and specificity of tNGS and mNGS. The results demonstrated that there was no statistically significant statistical difference in the sensitivity and specificity of the two methods, with P values of 0.248 and 0.290, respectively (**Tables 5**, **6**).

**Table 3:** Comparison of diagnostic performance of mNGS, tNGS and culture detection.

Methods	Positive number		Sensitivity (%)	Specificity (%)	F1 score	PPV	NPV
	Casual pathogen	non-causal pathogen					
mNGS	78	7	75.73	68.18	0.830	0.918	0.375
tNGS	99	9	96.12	59.09	0.938	0.917	0.765
Culture	15	0	14.56	100.00	0.254	1.000	0.200

*PPV and NPV represent positive predictive value and negative predictive value respectively.

**Table 4:** Diagnostic consistent of mNGS and tNGS detection methods.

Category/Result		mNGS		Total	Concordance rate (%)
		Causal pathogen	non-causal pathogen		
tNGS	Causal pathogen	78	30	108	70.40
	non-causal pathogen	7	10	17	
	Total	85	40	125	

**Table 5:** Sensitivity comparison of mNGS and tNGS detection methods.

Category/Result		mNGS		Total	χ2	P
		Causal pathogen	non-causal pathogen			
tNGS	Causal pathogen	74	25	99	1.334	0.248
	non-causal pathogen	4	0	4		
	Total	78	25	103		

**Table 6:** Specificity comparison of mNGS and tNGS detection methods.

Category/Result		mNGS		Total	χ2	P
		Causal pathogen	non-causal pathogen			
tNGS	Causal pathogen	4	5	9	1.119	0.290
	non-causal pathogen	3	10	13		
	Total	7	15	22		

**Figure 2:**
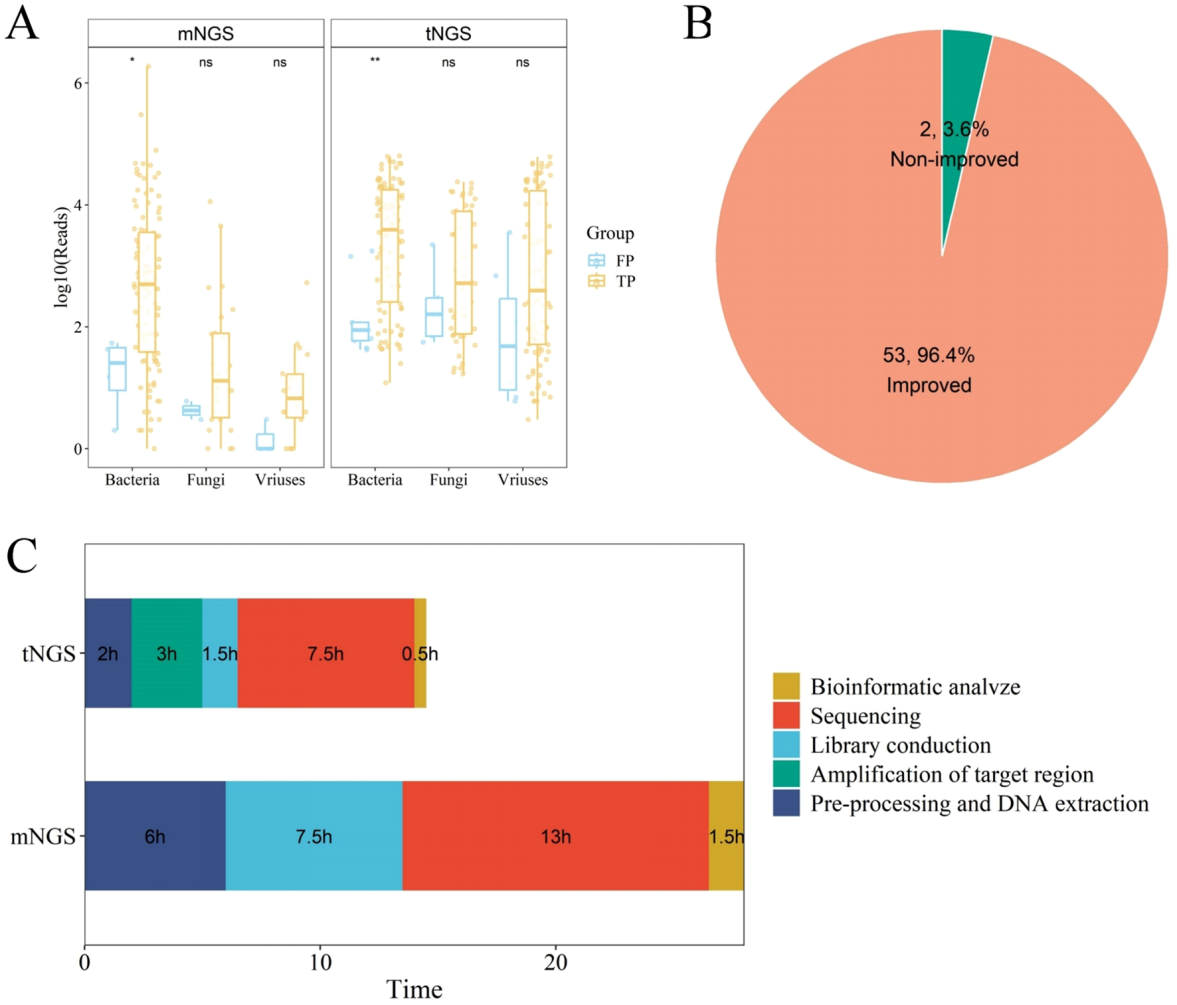
Evaluation of Clinical Diagnostic Efficacy of mNGS and tNGS Methods. **(A)** Box plot showing the log10(reads) in the true positive (TP) group compared with that in the false positive (FP) group. **(B)** Pipe chart revealing the improvement of 55 patients adjusted the anti-infective treatment. **(C)** Comparison of turnaround time between mNGS and tNGS.

Samples categorized into true positive (TP) and false positive (FP) groups were further compared based on either mNGS or tNGS. For mNGS method, 78 samples were regard as TP, while 7 samples were classified as FP. For tNGS method, 99 and 9 samples were grouped into TP and FP, respectively. A comparative analysis of the sequence counts for specific microbial types in TP and FP groups revealed that the count of bacterial sequences in TP samples was significantly higher than that in FP samples. Similarly, the counts of fungal and viral sequences also exhibited this pattern (**Figure 2A**). This suggests that the quantity of microbial sequences could be considered as a criterion for determining the authenticity of pathogenic infection.

A review of the treatment records of 125 patients with lung infections revealed that 55 patients had their anti-infective treatment adjusted based on the results of the NGS test. Among these 55 patients, 53 (96.4%) experienced a significant clinical improvement, whereas 2 (3.6%) did not respond to the treatment (**Figure 2B**; **Supplementary Table 2**). Additionally, the NGS test results of 47 patients were concordant with the initial clinical assessments, allowing the continuation of the initial anti-infective treatment regimen. The treatment plans of two patients were altered for reasons not associated with NGS test results, and no changes were made for 21 patients whose pathogens were not detected by NGS. Moreover, the average testing duration for tNGS was 14.5 hours, compared to 28 hours for mNGS (**Figure 2C**). Compared to mNGS and traditional culture methods, tNGS offers a swift and reliable turnaround time (TAT), which can aid physicians in delivering more accurate anti-infective treatments to patients.

## 4 Discussion

With the emergence of new pathogens and the increasing drug resistance of pathogens, it is difficult to meet the needs of disease diagnosis and treatment with the traditional clinical pathogen detection methods, and there is an urgent need for new faster and more sensitive pathogen detection methods to guide clinical diagnosis and treatment. Recently, the utilization of mNGS in clinical settings has progressively gained traction. Numerous reports have highlighted its superiority over conventional detection methods. However, it has its limitations, such as the requirement for DNA and RNA separation, susceptibility to interference from host nucleic acids, and a comparatively high cost (Diao et al., 2021; Moragues-Solanas et al., 2021). On the contrary, tNGS combines the advantages of ultra-multiplexed PCR amplification with high-throughput sequencing, allowing for targeted sequencing of specific pathogenic microorganisms. This approach boasts advantages such as cost-effectiveness, customizability, and a shortened detection timeframe. The objective of this study was to comprehensively assess the diagnostic efficacy of both mNGS and tNGS in BALF samples, ultimately providing a valuable reference for clinical practice.

In this study, we conducted a retrospective analysis of the outcomes from mNGS and tNGS tests performed on BALF samples from 155 patients suspected of having lung infections, with a focus on understanding the technical differences and clinical applications of these next-generation sequencing technologies. Among the microorganisms ultimately identified as causative agents, the three most commonly detected bacteria were *Pseudomonas aeruginosa*, *Haemophilus influenzae*, and *Streptococcus pneumoniae*. This prevalence may be attributed to the high incidence of comorbid underlying diseases and the advanced average age of the patients in our study. Previous research has indicated that in elderly patients or those with underlying diseases, the pathogens responsible for lung infections are predominantly gram-negative bacteria, such as *Pseudomonas aeruginosa* and *Klebsiella pneumoniae* (Han et al., 2018; Zhang et al., 2023). As for fungal pathogens, the most commonly identified species include *Candida albicans*, *Pneumocystis jirovecii*, and *Aspergillus fumigatus*, findings that are consistent with relevant epidemiological studies (Peng et al., 2018; Li et al., 2021). The top three DNA viruses detected were EBV, *cytomegalo virus*, and *human herpes virus 7*, with their distribution patterns mirroring those reported by Liu et al (Liu et al., 2022). Notably, the number of EBV strains detected by tNGS was significantly higher than that by mNGS (31 vs. 12 strains), but most were considered to be part of the respiratory tract’s normal flora.

Except *Epstein-Barr virus*, Gram-negative bacilli such as *Haemophilus influenzae*, *Klebsiella pneumoniae*, and *Pseudomonas aeruginosa* may exist as colonizing organisms. Their colonization patterns are typically associated with host immune status, environmental exposures, and microbiome interactions, particularly in immunocompromised individuals or those with prolonged hospitalization (Man et al., 2017). In immunocompetent populations, *Pneumocystis jirovecii* colonization is frequently observed in the absence of overt clinical symptoms or imaging abnormalities (Aguilar et al., 2021), and clinicians should comprehensively evaluate clinical context to determine their pathogenic role. The disparity in detection rates between mNGS and tNGS might be explained by the fact that mNGS sequencing necessitates the elimination of host background genes, which could impact its detection efficiency for gram-negative bacteria, viruses, and intracellular bacteria (Thorburn et al., 2015; Zhou et al., 2019). Furthermore, this result underscores the exceptional sensitivity of tNGS in detecting viruses. Comparative analyses revealed that mNGS and tNGS exhibited similar detection capabilities for various types of respiratory pathogens, both surpassing the traditional culture method in effectiveness, in line with the findings of Yin et al. (Yin et al., 2024). However, it was also concluded that mNGS demonstrated significantly superior performance than tNGS in identifying pathogenic respiratory pathogens within BALF samples (Simner et al., 2018).

An in-depth analysis of the distribution patterns of pathogens across diverse age groups revealed that, in patients aged 65 and above, the predominant pathogens were *Pseudomonas aeruginosa*, *Streptococcus pneumoniae*, *Klebsiella pneumoniae*, and *Candida albicans*. Conversely, *Parvimonas micra*, *Haemophilus influenzae*, *Mycoplasma pneumoniae*, and *Bordetella pertussis* were more prevalent among patients younger than 65 years. Prior research has established that, in the context of acute respiratory infections, *Klebsiella pneumoniae* and *Pseudomonas aeruginosa* exhibit an age-dependent distribution, with positive detection rates escalating with advancing age. These findings are in concordance with the outcomes of the present study (Prina et al., 2015; Cassini et al., 2019; Hu et al., 2020).

Regarding diagnostic efficacy, the sensitivity of tNGS was found to be superior to both mNGS and culture methods, with a sensitivity of 96.1% compared to 75.7% for mNGS and 14.6% for culture. However, in terms of specificity, the culture method demonstrated the highest specificity at 100.0%, followed by mNGS at 68.2% and tNGS at 59.1%. It is noteworthy that the statistical difference in sensitivity and specificity between mNGS and tNGS was insignificant. The results indicated that mNGS and tNGS exhibited notable advantages compared to BALF culture in detecting a wide range of pathogens, albeit with a lower specificity than the culture method. Numerous studies have been conducted to validate the superiority of mNGS and tNGS over traditional culture techniques in BALF samples (Wu et al., 2020; Liu et al., 2022; Yin et al., 2024), particularly for pathogens that are recalcitrant to cultivation.

A comparative analysis of the sequence counts of specific microbial types within the TP and FP groups, utilizing mNGS and tNGS assays, has shown that TP samples exhibit significantly higher sequence counts of bacteria, fungi, and viruses compared to FP samples. It is evident that a heightened sequence count is indicative of a greater likelihood of infection and can serve as a marker for clinicians in determining the underlying pathogens. However, it is important to note that the level of pathogen sequence count is influenced by a multitude of factors. Even if two samples exhibit an equivalent number of detected pathogen sequence counts, the actual concentration of the pathogens may vary. Consequently, sequence counts cannot be solely utilized as a definitive criterion for judgment. Rather, they must be interpreted in conjunction with the types of microorganisms detected, the clinical manifestations presented by the patients, and the outcomes of other laboratory tests.

Among all patients diagnosed with pulmonary infections, a substantial proportion of 44.0% (55/125) adjusted their anti-infective treatment regimens based on the outcomes derived from NGS. Notably, an overwhelming 96.4% (53/55) of these patients exhibited improvements in their clinical symptoms following the adjustment. The incorporation of NGS technology has markedly enhanced the precision of pathogen detection, a factor that is indispensable for the effective guidance of clinical treatment strategies. Moreover, tNGS boasts a significantly shorter average testing duration than mNGS. This advantage allows physicians to swiftly identify potential pathogens in patients, enabling more precise and efficient tailoring of treatment plans.

In summary, the clinical diagnostic efficacy of tNGS is comparable to that of mNGS in the context of clinical pulmonary infections. While tNGS does not fully supplant mNGS in identifying emerging and rare pathogens, it is significant to note that the predominant pathogens involved in clinical pulmonary infections are common ones, effectively detectable by tNGS. tNGS possesses the capability to detect both DNA and RNA simultaneously, exhibiting high sensitivity for pathogens present in low abundance within samples. Consequently, tNGS emerges as a novel solution for detecting the pathogens of clinical pulmonary infections.

However, it is imperative to acknowledge several limitations of this study: Firstly, as a retrospective single-center study, the inclusion of a relatively small sample size may introduce potential biases in the results. Secondly, RNA virus sequencing was not performed using mNGS, thereby limiting the ability to assess the diagnostic efficacy of mNGS specifically for RNA viruses. Lastly, the interpretation of the results obtained from both mNGS and tNGS relies heavily on the clinician’s combined judgment of the patient’s clinical presentation and the test results, which introduces a degree of subjectivity in the interpretation process.

## Data Availability

The datasets presented in this study can be found in online repositories. The names of the repository/repositories and accession number(s) can be found below: https://ngdc.cncb.ac.cn/gsa, CRA021644.
